# Three-dimensional reconstruction of root shape in the moth orchid *Phalaenopsi*s sp.: a biomimicry methodology for robotic applications

**DOI:** 10.1186/s13104-018-3371-0

**Published:** 2018-04-25

**Authors:** Anand Kumar Mishra, Andrea Degl’Innocenti, Barbara Mazzolai

**Affiliations:** 1 Center for Micro-BioRobotics, Istituto Italiano di Tecnologia (IIT), Viale Rinaldo Piaggio 34, 56025 Pontedera, Pisa Italy; 2 The BioRobotics Institute, Scuola Superiore Sant’Anna (SSSA), Viale Rinaldo Piaggio 34, 56025 Pontedera, Pisa Italy

**Keywords:** Photogrammetry, 3D printing, 3D reconstruction, Biomimicry, Bioinspiration, Bioinspired robotics, Plant-inspired robot, Root, Orchid, *Phalaenopsi*s

## Abstract

**Objective:**

Within the field of biorobotics, an emerging branch is plant-inspired robotics. Some effort exists in particular towards the production of digging robots that mimic roots; for these, a deeper comprehension of the role of root tip geometry in excavation would be highly desirable. Here we demonstrate a photogrammetry-based pipeline for the production of computer and manufactured replicas of moth orchid root apexes.

**Results:**

Our methods yields faithful root reproductions. This can be used either for quantitative studies aimed at comparing different root morphologies, or directly to implement a particular root shape in a biorobot.

**Electronic supplementary material:**

The online version of this article (10.1186/s13104-018-3371-0) contains supplementary material, which is available to authorized users.

## Introduction

There are many methods for the three-dimensional (3D) reconstruction of objects: they are mainly based on image, laser or X-ray scanning technologies. Laser scanning (LS) is one of most popular tools for 3D reconstruction. LS has several applications, such as reconstructing buildings and heritage sites, landscape monitoring, object acquisition for reverse engineering or inspection, and in robotics (e.g. for the exploration or digitalization of indoor environments) [1–4]. Apart from observation and construction, LS methods have also been used in biology, including morphological studies in plants (for instance cereals and saffron) [5–7].

Albeit LS represents the gold standard in terms of accuracy and yields solid models, it also displays some limitations: it does not provide color-textured information, requires costly equipment, high maintenance, and generates fuzzy points over highly textured and reflective surfaces [8]. Therefore, photogrammetry (or image scanning, IS) got strong recommendations by engineers and scientists, due to cheaper cost and low infrastructural needs. The two methods can also be combined when needed, cf. [9–15]. X-ray scanning is not as vastly explored as LS or IS, since it cannot be used for outdoor applications and it is usually very expensive; still, there are few studies on plants phenotype or root tomography [16, 17]. A recent and completely different approach to 3D shape acquisition is *dip transform*, where the object is reconstructed by soaking it in water at different orientation and each time measuring water displacement [18].

IS has been used for several research applications in a wide variety of disciplines: Drap and McCarthy et al. developed image-based scanning methods for underwater archaeological studies [19, 20]; Shashi et al. demonstrated 3D modeling and visualization of buildings by photogrammetry; moreover, Thali et al. used IS for forensic analyses [21], and Paul Siebert et al. adopted the same approach for human body 3D reconstruction [22]. Biological instances of photogrammetry comprise botanical inquires; Li et al. studied the digitization and visualization of tomato plants in indoor environments by using the stereo sensor Microsoft Kinect [23], while Nguyen et al. reconstructed different plants (cabbage, cucumber and tomatoes) through high quality images, taken at different angles through a stereo camera [24]; a 3D phenotyping of rose was undertaken by Chéné et al. using a depth camera [25]. Finally, Paproki et al. discussed a new method of IS plant analysis based on mesh processing technique [26]. To the best of our knowledge, however, no studies focused on shape acquisition of individual root apexes via IS.

Apart from fundamental research, the problem is interesting for plant-oriented biorobotics: digging robots inspired by plants typically mock the biological feature of roots of penetrating from the tip, cf. [27]; consequently, apical geometry has to be suitable for soil penetration, and might be optimized to different environmental conditions. Real roots can offer valuable examples of well-performing root shapes.

In this paper we propose an IS-based pipeline for the 3D reconstruction of root apexes. While a more comprehensive investigation of shape in different species—encompassing different ecological or physiological conditions—remains to be faced, here we approached the matter on a single model species (the moth orchid), chosen for having aerial and macroscopic root apexes.

We took sets of micrographs with varying orientation from a number of root tips. For each sample, we then computer-generated 3D models. We finally 3D-printed faithful replicas at different scales. Although significant tuning will be needed for different species, our method provides the groundwork for future studies. It is also worth mentioning that the pipeline produces high-resolution prototypes (unusual for photogrammetry), and produces textured in silico models.

## Main text

### Methods

#### Plants

**Fig. 1:**
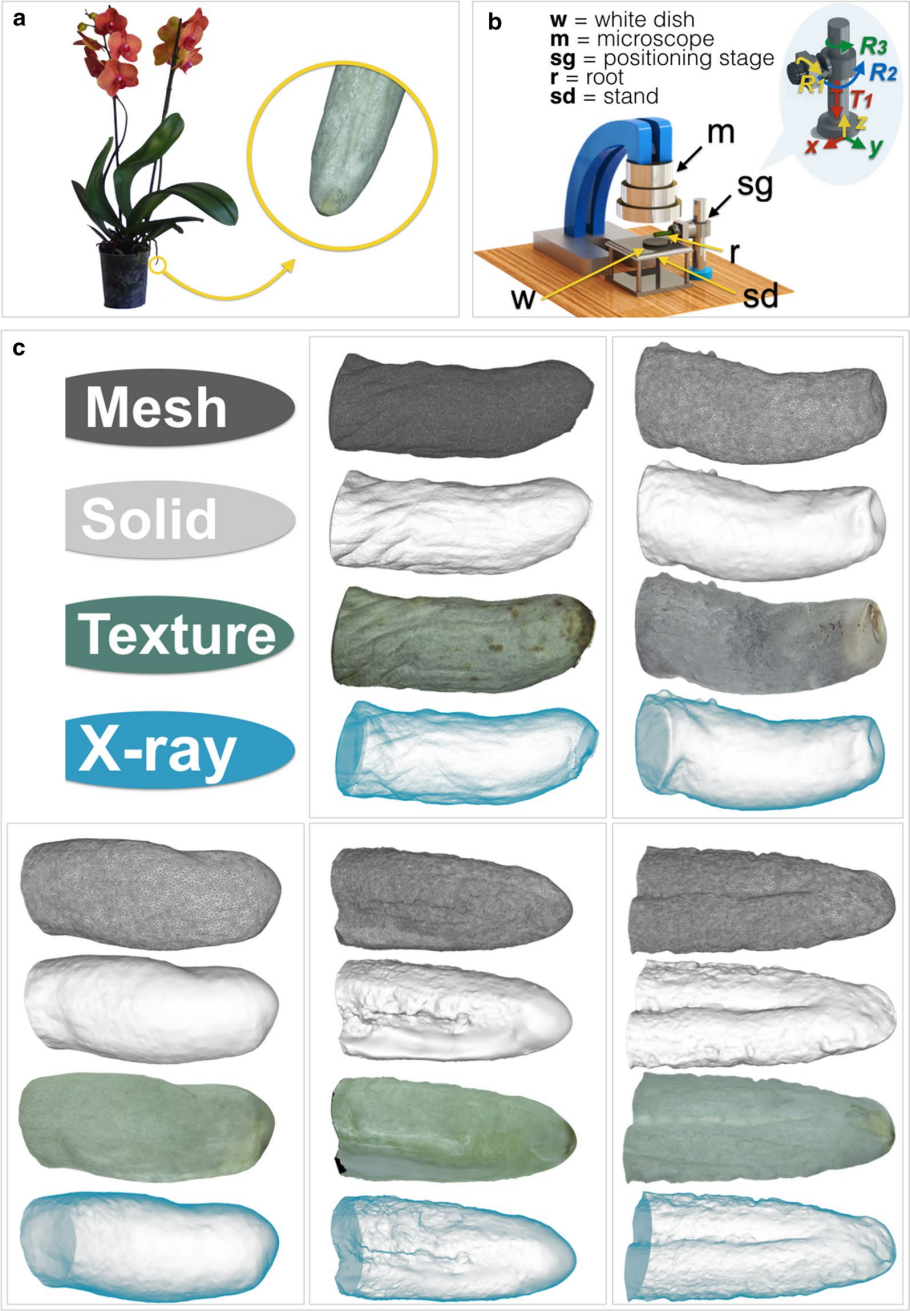
Generating computer models for five orchid root apexes. **a** Overall appearance of a moth orchid (*Phalaenopsis* sp.) commercial hybrid; the yellow circle contains a magnification of a root apex. **b** Simplified representation of the setup for the production of image sets; groups of micrographs at different views are taken by systematically adjusting root position thanks to a positioning stage. The comic (top, right) details on the four degrees of freedom (*R*_*1*_, *R*_*2*_, *R*_*3*_ and *T*_*1*_) yielded by the stage. **c** Snapshots of three-dimensional computer models for the five roots reconstructed, viewed in mesh, solid, textured, and X-ray modes

For the main root shape analysis, we used commercial moth orchid hybrids (*Phalaenopsis* sp., Orchidaceae Epidendroideae, Fig. 1) purchased from local flower shops. Specimens were kept in a growing chamber (Seed germinator SG 15, Nuova Criotecnica Amcota) at 25 °C, with 70% relative humidity and a light cycle of 12/12 h. A total of five different roots were included in the study; these were selected on the basis of straightness and overall healthy appearance. Root apexes were cut; sniped roots were measured (length and diameter), then immediately imaged. A preparatory study (see “Pre-analysis”) was conducted on a single zucchini fruit (*Cucurbita pepo* var. *cylindrica*, Cucurbitaceae) brought from the supermarket and promptly used. Its size in terms of length and diameter was also recorded. The zucchini was chosen as an example of a plant organ of macroscopic scale with complex texture and ideal shape.

#### Pre-analysis

Before starting root analysis, we performed the primary test on the zucchini. We took 250 images of the zucchini with a reflex camera (Canon EOS 550D, with an EFS 18–55 mm objective): a first group of 220 longitudinal photographs were obtained by rotating the fruit 360° around its main axis; 30 additional images were taken at varying heights. All pictures were used to generate a solid model. The detailed description about shape generation method is described in “Image processing” section.

#### Imaging

Each root was cut, then brought under a microscope to get high-resolution and high-texture images. We developed a setup consisting of the microscope (Hirox KH-7700 Digital Microscope System, with AD5040LOWS lens), a sliding stand with white disks for setting image background, and a positioning stage to rapidly change root orientation (Fig. 1). Such device is actually a combination of two different stages, that together yield four degrees of freedom, i.e. *R*_*1*_, *R*_*2*_, *R*_*3*_ and *T*_*1*_; first one is a rotary-type stage (Thorlabs CR1-Z7 Motorized Continuous Rotation Stage), used to hold the root from its proximal end and to rotate it around its main axis (*R*_*1*_, *x axis*). This rotary stage is highly precise, offering 360° continuous rotation and closed feedback motorized servo controller with 2.19 arcsec minimum incremental motion. For controlling the motor, we used the software Thorlabs Kinesis. The second stage is manual with three degrees of freedom, two rotations (*R*_*2*_ about the *y* axis, *R*_*3*_ about the *z* axis) and one translation (*T*_*1*_ along *z* axis). We took a total of 250 images per sample under white light, of which 220 about *R*_*1*_ (for 360°) at *R*_*2*_ = 0, 10 about *R*_*1*_ at *R*_*2*_ = 5°, 10 about *R*_*1*_ at *R*_*2*_ = 10° and 10 about *R*_*1*_ at *R*_*2*_ = 15°.

#### Image processing

Image sets were uploaded on a desktop computer (Intel Core i7-5960X 3.0 GHz processor, with 32 Gb RAM and graphics processing unit NVIDIA 358.91 GeForce GTX 980); shapes were reconstructed with Autodesk Remake 2017 software. Procedures include the generation of a cloud of points, a mesh, a solid shape and finally the addition of texture information. Holes were filled with extrapolation techniques.

#### Additive manufacturing

Filled solid models were used to fabricate mimicked roots by additive manufacturing technology (3D Systems ProJet HD 3000) using acrylic resin (3D Systems VisiJet M3 Crystal) material. To show scaling capability, artificial shapes were printed at different scales (i.e. 1:1 and 5:1).

#### Quality check

Through a scripting procedure, we measured root area for every single view of each set of root micrographs; then we took snapshots of each root virtual model at analogous orientations, and measured their root area as well. By doing so we obtained couple of values (real vs. reconstructed root areas), each corresponding to one out of more than two hundreds view angles within 360° of rotation. Data were plotted as line charts.

### Results and discussion

The zucchini we used had a length of ~ 120 mm and a central diameter of ~ 40 mm. The five different orchid root fragments had an average length of 21 ± 0.63 mm, and an average diameter of 4.41 ± 0.53 mm measured at half length.

A faithful shape reconstruction of the zucchini was rapidly achieved (Additional file 1). Accurate in silico models were also obtained from the sets of micrographs taken for each root (Fig. 1, see also Additional file 2. Even the mere evaluation of root two-dimensional projections over a full rotation on its main axis highlights a good correspondence between real roots and their computer models (Additional file 3). Still, in our experience the tip portion of the root is actually the hardest to reconstruct, likely due to the steeper angle of its profile, its smaller size and possibly its comparatively reduced complexity in terms of color texture. We believe, however, this is a limitation that several conceivable alternative methods would share.

As an epiphytic orchid, *Phalaenopsis* offers undoubtedly relevant technical advantages: its roots are exceptionally thick, and they do not need to be dug up from the soil (i.e. they are *aerial*). When cut, they keep their shape long enough for entire picture datasets to be taken without need for fixation; their surface complexity is adequate to allow reconstruction. Generally speaking, *Phalaenopsis* met our expectations for a *taxon* suitable for setting up a basic framework.

Other species will commonly display thinner and more flaccid roots, often with homogeneously diaphanous or whitish appearance. In order to transfer our pipeline to such plants—we envisage—some effort will be needed, mainly for the implementation of ad hoc fixation methods and expedients to increment surface information; in addition, it is worth considering that problems related to the recovery of root samples from the soil may arise for specific digging plants.

In some cases, and especially when working with model species, it would be interesting to test our procedure on whole mount preparations of stained root apexes (e.g. following in situ hybridization or immunostaining); this would aid 3D reconstruction, and eventually would yield colored virtual models combining shape and molecular information. For instance, one could assess both geometry and boundaries of an *elongation zone* (a fairly distal region of a root) by evaluating the expression domain of a suitable marker gene.

**Fig. 2:**
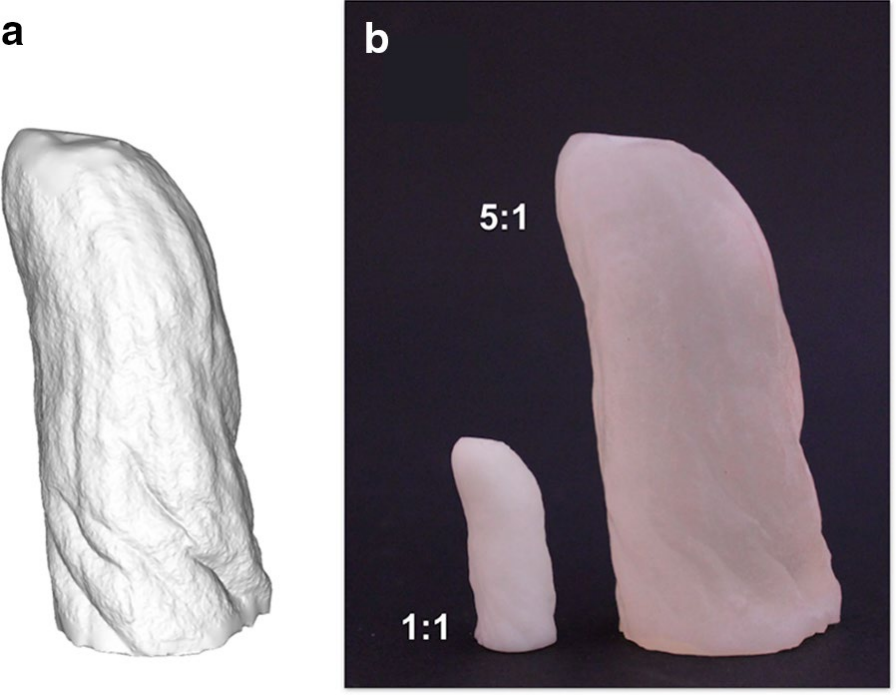
Additive manufacturing of an orchid root replica. **a** A snapshot of the solid-mode computer model for one of the reconstructed roots. **b** Fabricated replicas of the same root, pictured from roughly the same angle; as indicated in the picture, sample sizes are (left to right) 1:1 and 5:1 ratio with respect to their biological counterpart

Once filled, our computer models were 3D-printed. Whilst color is currently not implemented for manufacturing, size information is kept, and printed replicas are scalable (Fig. 2).

### Conclusion

We presented a new methodology to finely reproduce root shape. Our computer reconstructions mimic not only root morphology, but also texture; 3D-printed copies mimic solely the shape, in a scalable manner. As far as we can tell, this is the first pipeline that utilizes photogrammetry and 3D printing to replicate individual root anatomy.

This work focuses on a single species, chosen for having an easily approachable root apparatus: therefore, our primary interest is purely to open the way to future studies that either attempt to quantitatively assess the biological importance of different apex profiles in determining the digging performances of plants, or directly to implement a particular root shape in a root-inspired robot, cf. [27–30].

Beyond robotic applications, our study might ultimately contribute to basic biological inquires. In fact, virtual models could be used for long-term storage and sharing of data regarding morphology and staining patterns, cf. [31].

## Limitations

- Substantial tuning will be necessary to adapt our method to other species.
- Species displaying small, perishable or colorless root apexes will be more difficult to approach.
- Plants that possess digging roots will ultimately be of primary interest for the implementation of root-inspired digging robots; depending on the chosen *taxon*, issues related to the recovery of root apexes might be encountered.
- Resolution is typically lower at the very tip of the root.
- At present, 3D-printed roots do not reproduce color information.

## Additional files

**Additional file 1.** Three-dimensional reconstruction of a zucchini. Sample video of the virtual reconstruction of a zucchini fruit (*Cucurbita pepo* var. *cylindrica*), viewed in mesh, solid, textured, and X-ray modes.

**Additional file 2.** Visual comparison of real and 3D-reconstructed roots. Sample video of micrograph set and three-dimensional (3D) reconstruction (viewed in mesh, solid, textured, and X-ray modes) for each of the five orchid root apexes of the study.

**Additional file 3.** Variation in the two-dimensional projections of real roots and their virtual counterparts. The area occupied by a root in each of the micrographs taken varying the view around its main axis (a full rotation is displayed) is compared to the root area obtained from the corresponding screenshot of the virtual model.

## Abbreviations

3D: three-dimensional
IS: image scanning
LS: laser scanning
