# Supplementary figures and images for: Three-dimensional reconstruction of root shape in the moth orchid Phalaenopsis sp.: a biomimicry methodology for robotic applications
Source: BMC Res Notes. 2018 Apr 25;11:258. doi: 10.1186/s13104-018-3371-0 (PMC5918553; doi:10.1186/s13104-018-3371-0)

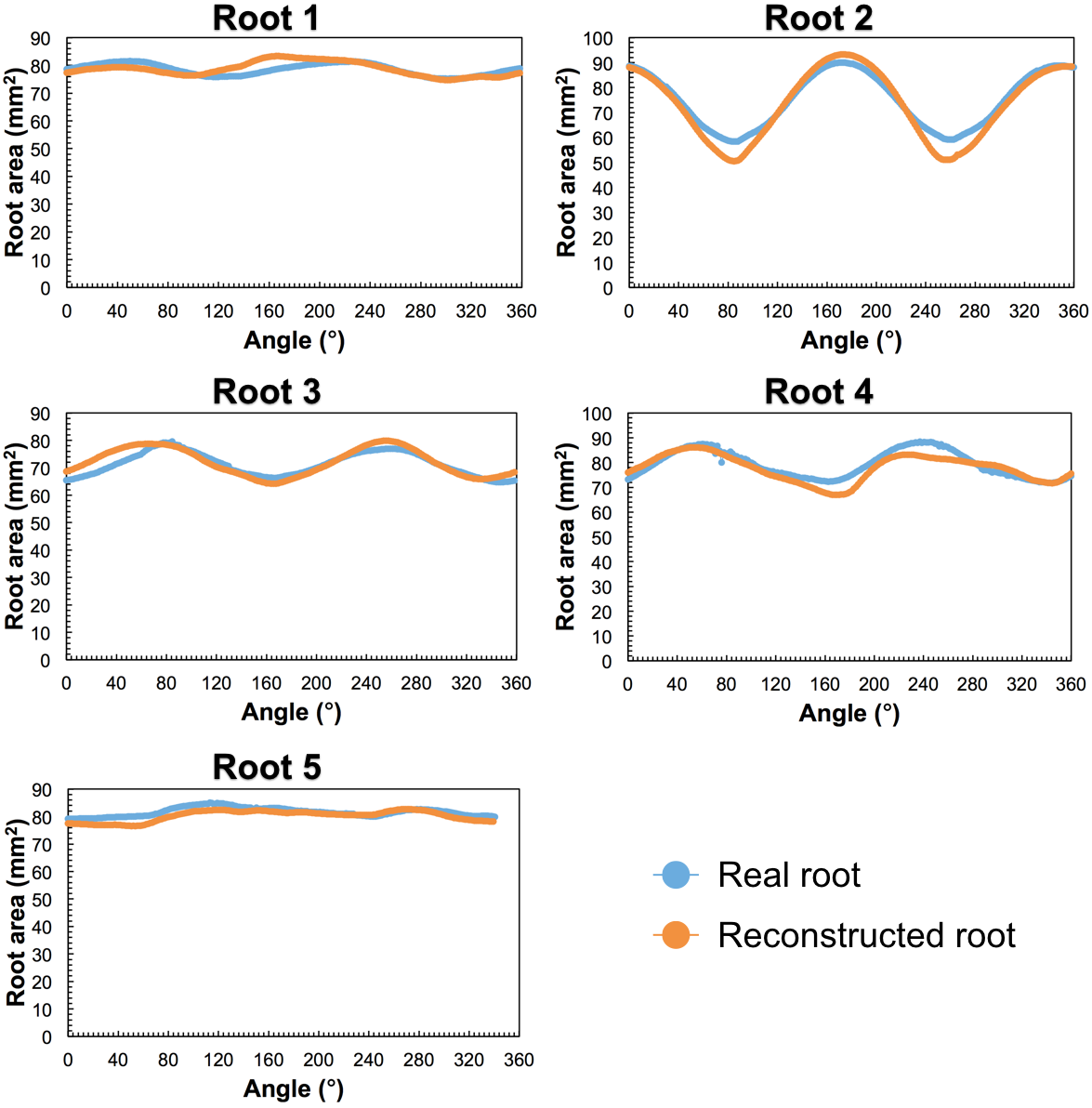

Supplement: Supplementary file 3 — Additional file 3. Variation in the two-dimensional projections of real roots and their virtual counterparts. The area occupied by a root in each of the micrographs taken varying the view around its main axis (a full rotation is displayed) is compared to the root area obtained from the corresponding screenshot of the virtual model. [file 13104_2018_3371_MOESM3_ESM.tiff]
